# Generalizable characteristics of false-positive bacterial variant calls

**DOI:** 10.1099/mgen.0.000615

**Published:** 2021-08-04

**Authors:** Stephen J. Bush

**Affiliations:** ^1^​ Nuffield Department of Medicine, University of Oxford, Oxford, UK

**Keywords:** false positive, variant calling, best practice, benchmarking

## Abstract

Minimizing false positives is a critical issue when variant calling as no method is without error. It is common practice to post-process a variant-call file (VCF) using hard filter criteria intended to discriminate true-positive (TP) from false-positive (FP) calls. These are applied on the simple principle that certain characteristics are disproportionately represented among the set of FP calls and that a user-chosen threshold can maximize the number detected. To provide guidance on this issue, this study empirically characterized all false SNP and indel calls made using real Illumina sequencing data from six disparate species and 166 variant-calling pipelines (the combination of 14 read aligners with up to 13 different variant callers, plus four ‘all-in-one’ pipelines). We did not seek to optimize filter thresholds but instead to draw attention to those filters of greatest efficacy and the pipelines to which they may most usefully be applied. In this respect, this study acts as a coda to our previous benchmarking evaluation of bacterial variant callers, and provides general recommendations for effective practice. The results suggest that, of the pipelines analysed in this study, the most straightforward way of minimizing false positives would simply be to use Snippy. We also find that a disproportionate number of false calls, irrespective of the variant-calling pipeline, are located in the vicinity of indels, and highlight this as an issue for future development.

## Data Summary

All analyses in this study use publicly available third-party software. All data and scripts necessary to replicate these analyses are provided within the article, through supplementary data files, and via the GitHub repository https://github.com/sjbush/FP_paper, which contains the benchmarking fasta, ‘truth set’ VCF and BED files. Sequencing data, for use with these benchmarking resources, was sourced from the FDA-ARGOS reference collection [1], and is available via BioProject accession PRJNA231221.

All supplementary material can be found at 10.6084/m9.figshare.14597145.

**Impact Statement**

This study contributes to the best practice literature on bacterial variant calling by providing guidelines for variant-call file (VCF) filtering. As there is flexibility within the VCF specification, one’s choice of variant-calling pipeline and choice of filter criteria are inextricably linked and so ‘universal filters’ – those applied routinely, irrespective of pipeline – should be avoided. This study characterizes all false-positive SNP and indel calls made by a broad range of pipelines, and identifies both the filters of particular efficacy and the pipelines to which they may most usefully be applied. In conjunction with a comparative performance evaluation of bacterial variant callers, it highlights those programs and filters which may best minimize false-positive calls.

## Introduction

Minimizing false positives is a critical issue when variant calling, particularly when the presence of a given variant can inform a clinical decision (for instance, when diagnosing disease [2] or disease susceptibility [3], or genotyping bacterial isolates [4]). While the choice of read aligner, genome to which reads are aligned, and variant-calling algorithm are all critical aspects of a variant-calling pipeline [5, 6], no method is without error. Errors are more likely when the reads contain contaminants [7], are divergent from the genome to which they are aligned [5] (or if this sequence is fragmented [8]), or if the genome from which they derive is hypermutable [9, 10]. Neither circumstance is uncommon when variant calling from bacterial sequencing data. As such, it is routine practice to post-process variant-call files (VCFs) using hard filter criteria intended to discriminate false-positive (FP) from true-positive (TP) calls [11–15]. Hard filters apply the simple principle that certain characteristics are disproportionately represented among the set of false-positive calls and that an empirically determined threshold can maximize the number detected. Machine-learning approaches to bacterial TP/FP classification, which could obviate this need for hard filters, are not yet widely available due to the lack of truth sets on which they may be trained.

The aim of this study was to identify which positional characteristics – that is, statistics recorded for each position, such as read depth – were disproportionately associated with bacterial FP calls and to produce generalizable recommendations for hard filters broadly applicable across a range of datasets. We stress that the recommendations made in this study are intentionally generic. We did not seek to optimize filter thresholds – these are invariably either caller- or dataset-dependent, or otherwise vary based on user requirements – but to instead draw attention to those filters of greatest efficacy and the pipelines to which they may most usefully be applied. This is because the choice of variant-calling pipeline and choice of filter criteria are inextricably linked. There is considerable flexibility within the VCF specification – variant callers differ in what is reported for each call, so there are different possibilities for filtering depending on the caller used. Recommendations for suitable filters must consider, therefore, the overall performance of each pipeline on bacterial data as well as what that pipeline includes in its VCF. Complicating this issue is that variant-calling solutions for eukaryotes are generally better-described but not necessarily appropriate for bacteria. For example, while previous studies have sought to optimize GATK filters [13, 16], it is important to note that GATK, and its best practice guidelines, were originally devised only for Illumina-centred human genome research [17].

To characterize the attributes of bacterial false-positive calls, we used an approach previously described [18], taking reads sequenced from one species with a closed genome, mapping them to another closed genome, calling variants, and then comparing this set of calls to the set of calls made using pairwise whole-genome alignment. We sourced sequencing data from the FDA-ARGOS reference collection [1], a public database of microbial genomes sequenced at high depth using both short- (Illumina HiSeq4000) and long-read (PacBio) technologies. Using this database, we curated six truth sets – data from the Gram-positive *Bacillus anthracis* and *Enterococcus faecalis *, Gram-negative *Escherichia coli *, *Francisella tularensis *, and *Salmonella enterica *, plus *Mycobacterium tuberculosis* – manually selected to represent a range of degrees of divergence between the sequenced reads and the genome to which they will be aligned (this being one of the principal determinants of variant-calling accuracy [5]), and a variant density spanning several orders of magnitude.

For each of these six genomes, we called variants using 166 different variant-calling pipelines. These pipelines comprise the combination of 14 short-read aligners [Bowtie2 [19], BWA-mem and BWA-sw [20], GASSST [21], GEM [22], HISAT2 [23], minimap2 [24], MOSAIK, NextGenMap, SMALT (http://www.sanger.ac.uk/science/tools/smalt-0), SNAP [25], Stampy, both with and without pre-alignment with BWA-aln [26], and Yara [27]] with up to 13 variant callers (DeepVariant [28], Freebayes [29], GATK HaplotypeCaller [30, 31], LoFreq [32], mpileup [33], Octopus [34], Pilon [35], Platypus [36], SNVer [37], SNVSniffer [38], SolSNP, Strelka [39] and VarScan [40]), plus 4 ‘all-in-one’ pipelines: Breseq [41], Snippy, SpeedSeq [42] and SPANDx [43]. The performance of each pipeline was evaluated on the basis of precision (positive predictive value), recall (sensitivity) and F-score, the harmonic mean of precision and recall [44], with true- and false-positive SNP and indel positions identified using the benchmarking tool hap.py (https://github.com/Illumina/hap.py). By plotting the distribution of various positional characteristics for the resulting set of true- and false-positive calls, we can highlight where the distributions differ. A VCF filter applied at this point could then effectively distinguish the two.

A key feature of this study is its scope. Variant callers that are, in general, higher-performing do not by definition generate a large number of false positives. We therefore needed a large number of VCFs in order to obtain enough false positives that we may characterize their distributions. Filter criteria derived on the basis of such a dataset would then have broad applicability, and would not necessarily be limited to any particular species or pipeline. However, somewhat paradoxically, a practically applicable set of VCF filter recommendations also requires a set of ‘good’ false-positive calls, those produced by pipelines that already score highly both for precision and recall. These are more likely to represent contemporary methodologies, and to be routinely used. Poorly performing pipelines will generate many false positives, but by virtue of being poorly performing are unlikely to be commonly used to begin with. Accordingly, we examine the characteristics of false-positive calls both for the entire dataset and for a subset of the most highly performing pipelines. We take this position because the aims of our study are pragmatic. We sought to characterize the errors of variant-calling pipelines that already perform reasonably well, as through VCF filtering the user may then improve them further. There is an unavoidable circularity to this approach: we first needed to evaluate the performance of multiple variant-calling pipelines in order to discard those that produce ‘too many’ false calls (whether positive or negative), reasoning that on this basis they would not be representative of a reasonable use case anyway. Why optimize such a pipeline when the data suggests you should have used a better one to begin with, and then optimize that? To that end, this study complements our previous performance evaluation of bacterial variant callers [5], and draws further attention to those which are particularly high-performing.

With regard to the false-positive calls, there are as many positional characteristics to examine as there are reported across the set of all variant callers. For practicality, we focused on nine characteristics generally applicable to multiple callers. These are:

1. Read depth, the total number of reads mapped at a given position. Different variant callers report the depth differently, with some reporting an absolute read count and others (such as GATK) only those reads which pass internal filters and that are actually used for variant calling. We also considered the total number of reads supporting the variant allele.
2. Variant-call quality (QUAL), the Phred-scaled probability that the variant call is correct. We also considered the ‘quality by depth’ (reported by GATK as ‘QD’), the average quality of each variant-supporting read. QD values normalize the variant quality in order to avoid misleadingly high values of QUAL brought about by deep coverage. For this reason, the GATK guidelines recommend that for filtering purposes it is better to use QD than either QUAL or read depth directly (https://gatk.broadinstitute.org/hc/en-us/articles/360035890471-Hard-filtering-germline-short-variants, accessed 28 January 2020).
3. Variant allele frequency, also known as allele balance, the proportion of reads that support the non-reference allele. In a VCF generated from a haploid genome, heterozygous loci (which have a variant allele frequency <1) are not expected, their presence suggesting either mapping errors among repetitive regions, recombination between genes on the chromosome and extra-chromosomal elements (the extent of which varies between species [45]), that the input reads are sourced from multiple strains of the same species [46], or that they are a mixture of closely related, but different, species.
4. Position of the reads with respect to the variants within them, considering both mapping location (whether reads map both up- and downstream of the call) and orientation (whether variant-containing reads map to both strands). Strand bias, where the alternate allele is disproportionately found on either the forward or reverse reads, increases the likelihood of a false-positive call [47]. Studies in cancer data suggest the efficacy of a ‘both strands’ filter, which requires a minimum number of reads mapping to both the forward and reverse strand [48, 49].
5. Distance to nearest variant call, either SNP or indel. Errors are not necessarily made in isolation, and variant calls in close proximity could indicate an underlying difficulty with calling in that region. Aligners map reads to the genome independently, but at any given locus the optimal combination of pairwise alignments (of each read against the genome) is not necessarily the optimal multiple sequence alignment (which would be used for variant calling) [50]. In these circumstances, many variant callers perform local realignment, although this is technically challenging around indels and in regions of low complexity. As such, we expect that false positive calls are more likely to be nearer to each other than to a true positive call.

We plotted the distribution of each of these nine characteristics for the pooled set of all true- and false-positive calls made across all six samples, as well as per pipeline. On the basis of these distributions we can highlight those filters of particular utility and draw generalizable conclusions about effective strategies for minimizing false-positive variant calls. The datasets used for this analysis may also be used to benchmark bacterial variant callers, and are available at https://github.com/sjbush/FP_paper.

## Methods

### Variant-call truth sets

To examine the characteristics of false-positive variants, we first required a truth set of variant calls against which the output of any given variant-calling pipeline could be compared. To do so, we can obtain sequenced reads from one sample with a closed genome, align them to another closed genome, call variants from these alignments (using a range of pipelines), and then compare that set of calls to the set of calls made using pairwise whole-genome alignment. For our purpose, the latter set of calls constitutes the truth set. To generate these, we sourced data from the FDA-ARGOS reference collection [1] (BioProject accession PRJNA231221), a public database of microbial genomes for diagnostic use sequenced using short- (150 bp paired-end Illumina HiSeq4000) and long-read (PacBio RS11 P6-C4) technologies. We parsed the FDA-ARGOS collection to produce a longlist of 122 bacterial samples, each with (a) an unambiguous species annotation (i.e. we excluded those with names of the form ‘Genus sp.’), (b) paired sets of publicly available Illumina reads and an associated Illumina/PacBio hybrid assembly, (c) an NCBI assembly classification of ‘complete genome’ (i.e. the core genome – and that of plasmids, if present – was gapless, with no runs of 10 or more N bases, there were no unplaced scaffolds, and the assembly was not considered to have partial genome representation). The FDA-ARGOS assemblies were created using a pipeline comprising SPAdes [51], Canu [52], HGAP [53], Celera Assembler [54], Pilon [35], and manual curation, as previously detailed [1]. For each assembly, the corresponding short-read sequencing data was high depth, approx. 300x.

Further to quality checks (see below), we restricted analysis to a shortlist of six samples: *Enterococcus faecalis* (accession FDAARGOS_338), *Escherichia coli* (FDAARGOS_536), *Francisella tularensis* (FDAARGOS_598), *Salmonella enterica* (FDAARGOS_687), *Bacillus anthracis* (FDAARGOS_700) and *Mycobacterium tuberculosis* (FDAARGOS_751). For each sample we curated a quartet of files – fastq, fasta, VCF, and BED – as benchmarking resources. These files represent, respectively:

- the original FDA-ARGOS paired-end Illumina sequencing reads (for the aforementioned samples, the corresponding SRA run accession IDs are SRR5448651, SRR8180486, SRR8283296, SRR9163323, SRR9171533, and SRR9176751, respectively), randomly down-sampled to 1000000 reads using seqtk ‘sample’ v1.3 (https://github.com/lh3/seqtk) with seed 42,
- an NCBI reference genome, against which these reads will be aligned and variants called,
- a truth set of variant calls, to be contrasted with the pipeline VCF using the haplotype comparison tool hap.py (https://github.com/Illumina/hap.py),
- a list of higher-confidence regions within the reference genome, to be provided as input to hap.py using parameter -f.

This subset of FDA-ARGOS samples was handpicked to ensure that collectively the six species represented (a) a range of degrees of divergence between the sequenced reads and the genome to which they will be aligned, calculated using Mash v2.1 [55], and (b) a variant density spanning several orders of magnitude (from a ‘basic’ truth set containing 19 SNPs and 13 indels to a ‘complex’ truth set containing 83385 SNPs and 381 indels). Characteristics of the FDA-ARGOS samples are given in Table S1 (available in the online version of this article), with Mash distances relative to their reference genome varying from a negligible 2.38×10^−5^ (* Bacillus anthracis *) to 0.03 (* E. coli *).

We made whole-genome alignments between each FDA-ARGOS assembly and its corresponding NCBI reference genome using both nucmer [56] and paftools [24] with a range of parameters, then identified consensus calls within one-to-one alignment blocks. These consensus positions constituted the truth set for evaluation. All ambiguous positions – where the set of nucmer and paftools calls were discordant – were considered lower-confidence. For the purpose of comparing VCFs with hap.py (see below), only calls outside of these regions were counted; the location of these regions comprise the BED.

This workflow also comprised several quality-checking and validation steps:

(a) Selection of NCBI reference genome

We required at least one NCBI reference genome that was not the same as the FDA-ARGOS assembly. Most bacteria, but not all, have only one associated NCBI reference genome. As of December 2020, there were only three species in the FDA-ARGOS collection with >1 reference genomes: *Salmonella enterica* (Typhimurium str. CT18 and LT2), *Bacillus anthracis* (str. Ames and str. Sterne), and *E. coli* (K-12 substr. MG1655, O157:H7 str. Sakai, IAI39, O83:H1 str. NRG 857C, and O104:H4 str. 2011 C-3493). In these cases, we chose the first of the listed options.

(b) Soft-masking lower-quality loci in the FDA-ARGOS assembly

To identify lower-quality regions of each assembly, we re-mapped the corresponding Illumina reads using minimap2 v2.17 [24] with default parameters, and soft-masked (using BEDtools ‘maskfasta’ [57]) all bases where there was either no coverage or the most common nucleotide represented <99 % of the total depth at that position. We then used the Illumina reads to call variants in the masked assembly using Snippy v4.3.6 with default parameters. This was a negative control: as these reads were in principle sourced from the FDA-assembled genome (that is, they are essentially short fragments of it), we did not expect variants to be present. We therefore considered any variants detected by Snippy to be discordant base calls between the Illumina and PacBio reads, ostensibly reflecting sequencing errors. We masked these ‘discordant call’ positions in the same manner as the ‘low quality’ positions, above.

(c) Whole genome alignment of the soft-masked assembly to the NCBI reference

We used both nucmer v4.0.0 and paftools (packaged with minimap2 v2.17) to perform whole-genome alignment of the masked FDA-ARGOS assembly with the corresponding NCBI reference, standardizing the representation of variants in each VCF using the ‘pre.py’ module of hap.py with parameters --leftshift --decompose. As variant detection can be parameter-sensitive, we ran nucmer in default mode (which requires that anchor matches, used to seed the alignment, are unique in the reference genome) while varying the -c (minimum cluster length), -g (maximum gap size) and -b (break length) parameters, from 25 to 200 at increments of 10, 90 to 900 at increments of 90, and 200 to 400 at increments of 200, respectively (the default values of -c, -g, and -b are 65, 90, and 200, respectively). We also ran paftools with default options with the exception of simultaneously varying the -l and -L parameters (minimum alignment length to, respectively, compute coverage and call variants), from 25 to 200 at increments of 25. In total, for each pair of genomes, we generated a set of *n*=548 VCFs (540 nucmer and eight paftools). These were parsed using BCFtools ‘isec’ [33] to create two summary VCFs: a consensus (intersect) set of variants present in all *n* VCFs (using isec parameter ‘-n=*n*’), and an ‘ambiguous’ set of parameter-sensitive variants, those called in up to *n*-1, but not all, VCFs [using isec parameter ‘-n-(*n*-1)’]. Coordinates from the latter VCF were used to create a BED file, from which a complementary BED of ‘higher-confidence’ positions (those where either the same variant call, or no variant call, was made in every VCF) was created using BEDtools ‘complement’. This file was used as input to hap.py.

The set of fasta, VCF and BED files for each of the six samples is available at https://github.com/sjbush/FP_paper. The fastq files are available via the Sequence Read Archive, with accessions given above.

### Variant-calling pipelines

We used 166 different variant-calling pipelines, the pairwise combination of 14 read aligners [Bowtie2 [19], BWA-mem and BWA-sw [20], GASSST [21], GEM [22], HISAT2 [23], minimap2 [24], MOSAIK, NextGenMap, SMALT (http://www.sanger.ac.uk/science/tools/smalt-0), SNAP [25], Stampy, both with and without pre-alignment with BWA-aln [26], and Yara [27]] with up to 13 variant callers [DeepVariant [28], Freebayes [29], GATK HaplotypeCaller [30, 31], LoFreq [32], mpileup [33], Octopus [34], Pilon [35], Platypus [36], SNVer [37], SNVSniffer [38], SolSNP (http://sourceforge.net/projects/solsnp/), Strelka [39] and VarScan [40]], plus the four ‘all-in-one’ pipelines Breseq [41], Snippy (https://github.com/tseemann/snippy), SpeedSeq [42] and SPANDx [43], each run with default parameters. Note that not all aligners could be successfully paired with each caller, due to the technical requirements of each program, and that not all callers simultaneously call both SNPs and indels. These pipelines were employed in a prior evaluation of bacterial SNP callers, with scripts available at https://github.com/oxfordmmm/GenomicDiversityPaper, and command lines and technical notes on their operation previously described [5]. The programs used, including version numbers and sources, are detailed in Table S2.

For each pipeline, the resulting VCF was compared with the truth set VCF to identify true-positive (TP), false-positive (FP) and false-negative (FN) positions, and to calculate performance metrics including precision (positive predictive value), recall (sensitivity), and F-score (the harmonic mean of precision and recall, a summary metric that ranks overall performance on a scale from 0 to 1). Precision is calculated as TP/(TP+FP), recall as TP/(TP+FN), and F-score as 2x ([precision x recall]/[precision+recall]).

As the same variant can be accurately represented in multiple ways, all VCF comparisons, and the calculation of performance metrics, were made using hap.py v0.3.12, which regularizes VCF contents, with parameters --decompose --leftshift --engine=vcfeval --preprocess-truth --set-gt hom (the latter requiring alleles in the pipeline VCF to be on the same haplotype as that of the truth VCF). Performance metrics were calculated only for those positions where the VCF FILTER column, if populated, was ‘PASS’.

hap.py produces two output files: a summary of the high-level performance metrics, including total number of TPs, FPs and FNs, plus a VCF comparing positions between the truth (i.e. FDA-ARGOS) and query (i.e. pipeline) VCFs. From this comparison VCF we obtained the positions of each FP call. The purpose of doing so was to cross-reference these positions with the original pipeline VCF, which contains positional information that may be used for filtering (for instance, by requiring a minimum read depth at that locus or variant allele frequency). As our interest was in VCF filtering, we were concerned only with biallelic calls, those where the hap.py comparison VCF showed ‘NOCALL’ for that position in the truth VCF and ‘FP’ in the query VCF, with neither the reference nor variant base(s) in each VCF represented by an ambiguity character (i.e. other than A, T, C or G). We also discarded those FP SNPs which were part of a complex variant or multi-nucleotide polymorphism. This was because it would not be possible to unambiguously interpret the positional information reported at these sites. A consequence of restricting analysis to biallelic sites is that the number of FPs reported in the hap.py summary file will also be greater than the number extracted from the comparison VCF.

## Results and discussion

### Datasets used

For the purpose of benchmarking variant-calling pipelines, we first parsed data from the FDA-ARGOS reference collection [1] to generate six truth sets (Table S1). These truth sets constitute a broad range of bacterial genomes, both Gram-positive and Gram-negative, and contain between 19 and 83 385 SNPs, and 13 to 381 indels. For each of the six genomes, we used 1 million Illumina HiSeq reads (150 bp paired-end) to call variants using 166 different pipelines (Table S2). The overall dataset comprised 990 VCFs (165 pipelines * 6), and contained a total of 26 529 429 SNP and 463 386 indel calls, of which 1 545 957 (5.8 %) and 246 852 (53.2 %) were false positives (FPs), respectively (Table S3). The performance of each pipeline – assayed as precision, recall and F-score – is given in Table S3, and generally high (discussed further below). For the purpose of characterizing the attributes of true- and false-positive calls, we discuss SNPs and indels separately, noting that some pipelines – those employing LoFreq, SNVer, SolSNP, or VarScan – could only call SNPs.

### Empirical characterization of true and false-positive SNP calls

**Fig. 1.**
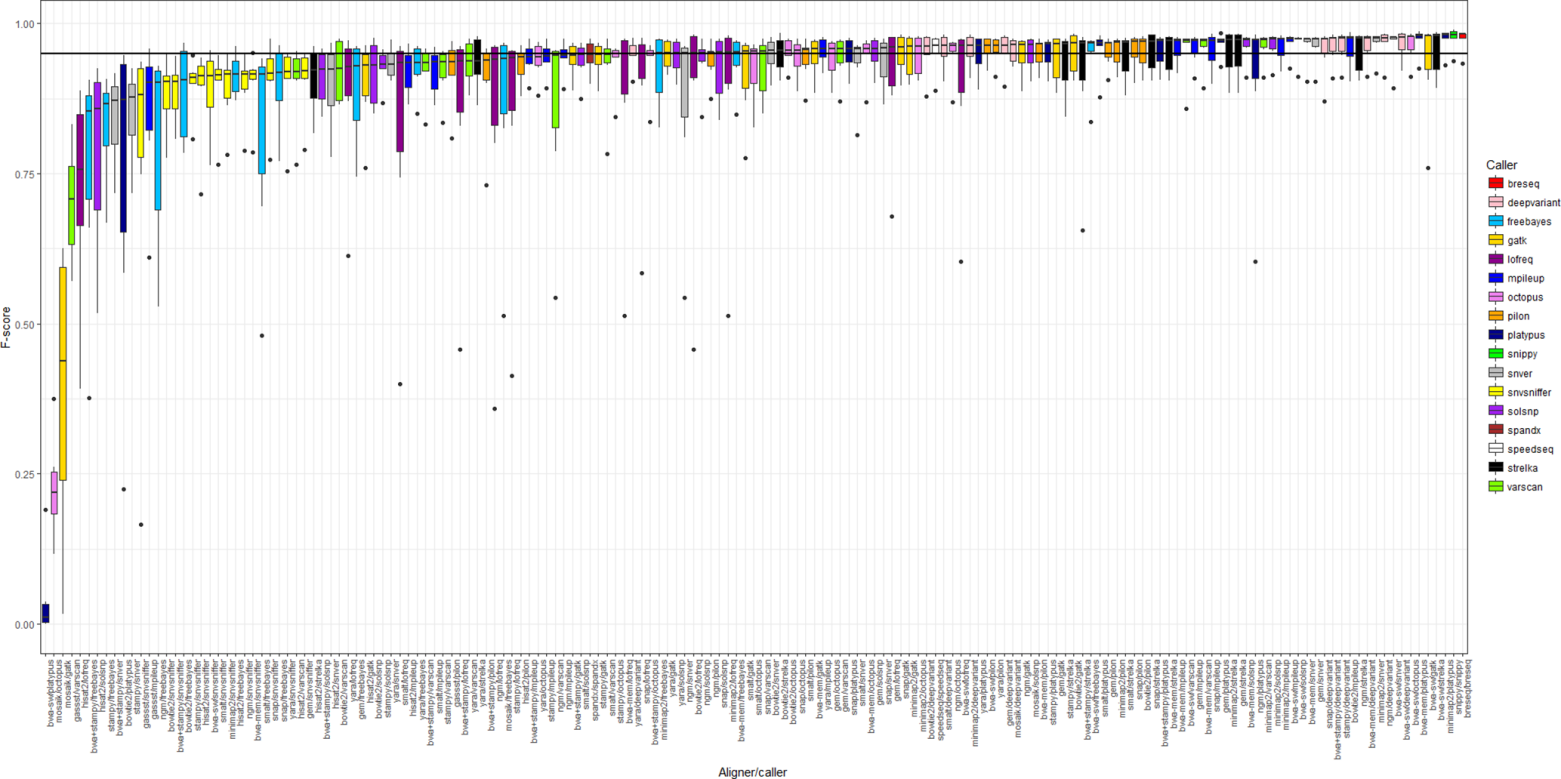
Median F-score for 166 SNP calling pipelines. Boxes represent the interquartile range of F-score, with midlines representing the median. Upper and lower whiskers extend, respectively, to the largest and smallest values no further than 1.5x the interquartile range. Data beyond the ends of each whisker are outliers and plotted individually. Pipelines are ordered according to median F-score and coloured according to the variant caller employed. The performance metrics for each pipeline, from which this figure was generated, are shown in Table S3. The line *y*=0.95, denoting a particularly high F-score, is marked.

Of the set of 990 VCFs, 479 (48 %) called SNPs with an F-score >0.95 (Table S3), as illustrated in Fig. 1 (comparable plots of precision and recall are given in Figs S1 and S2, respectively). Across the six samples, the three highest performing pipelines, with comparatively little variation in F-scores, were Breseq, Snippy, and minimap2/Platypus (Fig. 1). The set of pipelines with F-scores >0.95 were also enriched for pipelines using DeepVariant and Strelka, consistent with a previous performance evaluation of bacterial SNP callers [5].

To identify common characteristics of false, relative to true, positive SNP calls, we restricted analysis to a set of 23 683 336 biallelic TPs and 1 294 894 biallelic FPs (89 and 84 % of the total TP and FP calls, respectively), i.e. those where the SNP was not considered part of a complex variant or multi-nucleotide polymorphism. This was because we were concerned with the positional information – the contents of the VCF INFO and FORMAT fields – uniquely attributable to each SNP as it is on the basis of this that calls may later be filtered. Although the same SNP could be called as TP or FP by multiple pipelines, each pipeline makes its calls on the basis of different alignments and so populates these fields with different information. These paired sets of TPs and FPs therefore represent all (biallelic) TP and FP calls per pipeline, and so may count the same SNP multiple times.

**Fig. 2.**
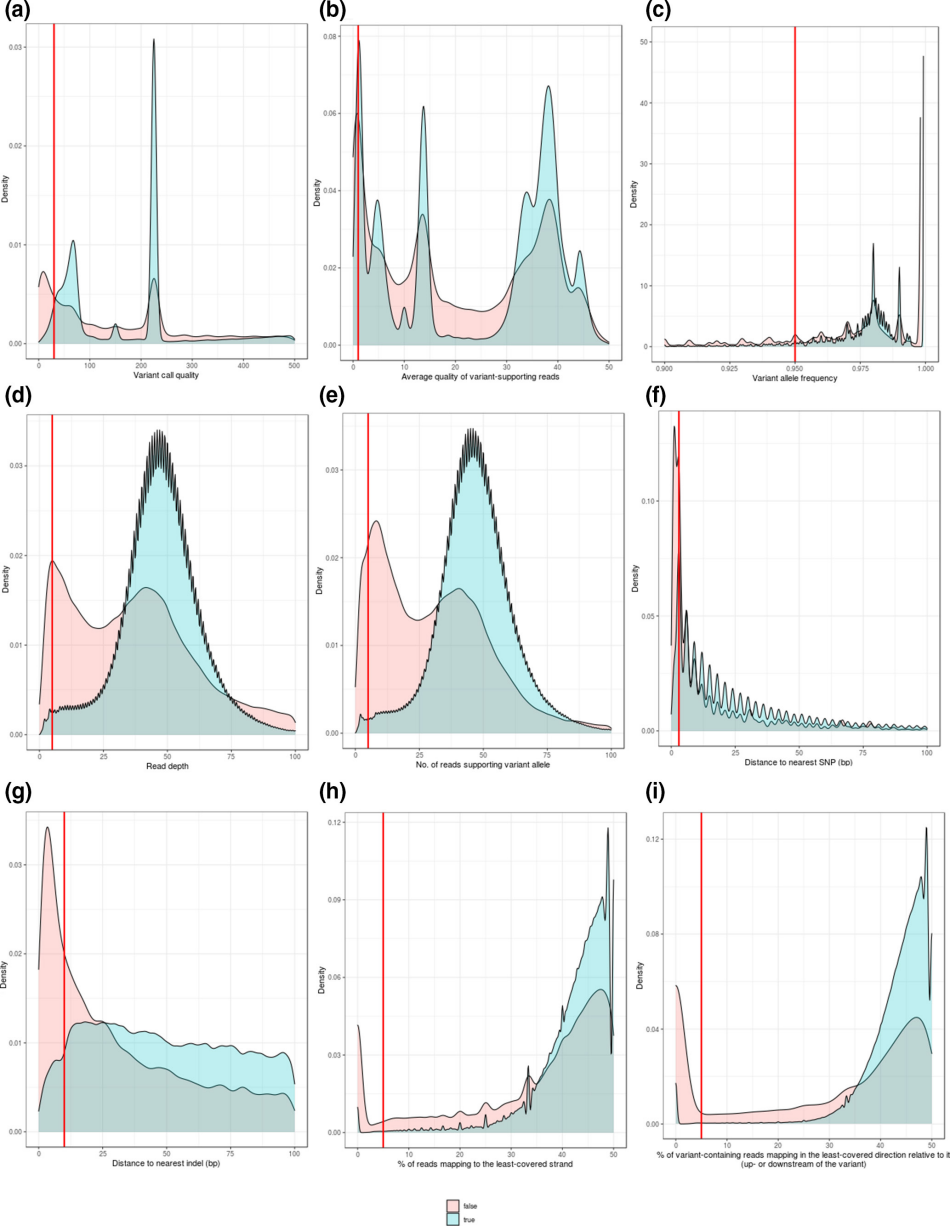
Characteristics of biallelic true**-** and **false-positive** SNPs. This figure shows data sourced from 990 VCFs, and represents 23 683 336 biallelic true positive and 1 294 894 biallelic false-positive calls. Density plots show the distribution of nine characteristics for the true (blue) and false (red) calls: (a) variant-call quality, (b) average quality per variant-supporting read, (c) variant allele frequency, (d) depth (total number of reads mapped at that locus), (e) number of reads supporting the variant allele, (f) distance to nearest SNP, (g) distance to nearest indel, (h) percentage of reads mapping to the least-covered strand, and (i) percentage of SNP-containing reads mapping in the least-covered direction away from it (in the latter two cases, 50 % indicates the variant is equally supported by reads on both the forward and reverse strand, and by reads mapping both up- and downstream of the variant, respectively). Red lines indicate potential hard filter criteria, empirically suggested. These criteria would discard SNPs that have: (a) variant-call quality ≤30, (b) average quality per variant-supporting read ≤1, (c) variant allele frequency ≤0.95, (d) read depth ≤5, (e) number of reads supporting the variant allele ≤5, (f) distance to nearest SNP ≤3 bp, (g) distance to nearest indel ≤10 bp, or ≤5 % of reads mapping to (h) the least-covered strand, or (i) in the least-covered direction (i.e. the variant is not proportionately supported by reads on both the forward and reverse strand, or both up- and downstream). A summary of the number of false-positive SNPs detected using these thresholds is given in Table 1. Note that data from SolSNP is not shown in plot A. This is because SolSNP does not follow the VCF specification for defining QUAL continuously, instead capping QUAL at 30. Note also that the ‘read depth’ distributions in plots D and E are not smooth because different variant callers calculate read depth differently, with some reporting absolute values and others an average (detailed in Table S4). A version of this figure restricted only to pipelines with F-score >0.95 is available as Fig. S3, showing quantitatively similar distributions with identical empirically derived filters. The set of distributions per pipeline are given in the Supplementary Archive.

Characteristics of the total set of TP and FP SNP calls are summarized in Fig. 2 and summarized in Table 1. Characteristics per pipeline are given in the Supplementary Archive. In general, however, FP SNPs have lower variant-call quality relative to TP calls, closer proximity to other variants (particularly SNPs), and are supported not only by fewer reads mapped to that locus and on either strand but with fewer variant-containing reads also mapped both up- and downstream. Generic hard VCF filter criteria may be suggested on the basis of the Fig. 2 distributions, noting that these are subjective and used here for illustrative purposes rather than as definitive guides to practice. These generic criteria would discard SNPs that have a variant-call quality ≤30, average quality per variant-supporting read ≤1, variant allele frequency (i.e. proportion of reads supporting the variant allele) ≤0.95, total read depth ≤5, number of reads supporting the variant ≤5, distance to nearest SNP and indel ≤3 and ≤10 bp, respectively, or ≤5 % of the reads mapping either to the least-covered strand or in the least-covered direction away from the variant (i.e. the variant is not proportionately supported by reads on both the forward and reverse strand, or both up- and downstream). These characteristics are not equally weighted, with some more informative than others. The most informative criteria – in terms of the percentage of FP calls detected – were proximity to the nearest SNP and variant-call quality, which by themselves could detect 39 and 27 % of the total FPs to which these filters could be applied, respectively (Table 1). One might expect that as bacteria are haploid, all heterozygous loci could immediately be dismissed as errors and that variant allele frequency would be the most efficacious filter. However, the data suggest that while filtering on the basis of variant allele frequency does indeed capture many FPs (25 % of the total FPs to which this filter could be applied), the majority of FPs were not mixed calls and that other filters had greater efficacy. It is important to emphasize that the distributions in Fig. 2 pool data from multiple pipelines, and that different pipelines have distinct error profiles. To that end, for some pipelines, it is not possible to cleanly distinguish the TP and FP distributions for a given characteristic (see Supplementary Archive) and so not all of the above filters can or should be applied, a point returned to below.

**Table 1.** Number of true- and false-positive variants detected using empirically determined hard filters

SNPs	Any one or more of the other criteria	Distance to nearest SNP <=3 bp	Variant-call quality <=30	Up-/downstream mapping bias (<=5 % of reads map up- or downstream of variant)	Variant allele frequency <=0.95	Avg. quality of variant-containing reads <=1	Strand bias (<=5 % of reads map to least-covered strand)	No. reads supporting variant <=5	Distance to nearest indel <=10 bp	Read depth <=5
Total no. of FP calls	1 294 894	1 294 893	967 140	94 608	1 137 831	711 545	691 565	978 302	760 877	1 294 828
No. of FP calls removed	821 144	509 391	260 965	25 258	288 645	75 558	68 990	90 049	60 064	94 623
% of FP calls removed	63.41	39.34	26.98	26.7	25.37	10.62	9.98	9.2	7.89	7.31
No. of FP calls only removed by this filter	na	199 322	54 816	5662	78 160	11 433	17 629	836	20150	0
% of FP calls only removed by this filter	na	15.39	5.67	5.98	6.87	1.61	2.55	0.09	2.65	0
Total no. of TP calls	23 683 336	23 683 334	18259439	1624305	20317662	14435763	12123579	18 258 302	15957 723	23683315
No. of TP calls removed	6558764	3853196	2272104	19366	286 053	807 924	75 565	132122	67755	205065
% of TP calls removed	27.69	16.27	12.44	1.19	1.41	5.6	0.62	0.72	0.42	0.87
Ratio of % of FP to % of TP removed	2.29	2.42	2.17	22.44	17.99	1.9	16.1	12.78	18.79	8.4
**Indels**	any one or more of the other criteria	distance to nearest indel <=10 bp	variant allele frequency <=0.95	strand bias (<=5 % of reads map to least-covered strand)	read depth <=10	distance to nearest SNP <=3 bp	variant-call quality<=20			
		
		
		
Total no. of FP calls	135 295	135 277	82 042	33 977	119 480	135 294	106 597			
No. of FP calls removed	114 314	86 324	31 135	8288	23 040	25 567	12 229			
% of FP calls removed	84.49	63.81	37.95	24.39	19.28	18.9	11.47			
No. of FP calls only removed by this filter	na	40 709	6961	1632	0	3844	1834			
% of FP calls only removed by this filter	na	30.09	8.48	4.8	0	2.84	1.72			
Total no. of TP calls	157 898	157 861	118 698	46 093	133 409	157 898	133 697			
No. of TP calls removed	41 150	5632	24 410	666	2879	10 597	1530			
% of TP calls removed	26.06	3.57	20.56	1.44	2.16	6.71	1.14			
Ratio of % of FP to % of TP removed	3.24	17.87	1.85	16.94	8.93	2.82	10.06			

In any case, incorporating this complex set of variables into a single mechanistic framework is difficult, as it requires we understand how each variable influences the likelihood that a variant is real as well as the interactions between them [58]. It is neither desirable nor realistic that a human should do this as the task is better suited to machine classifiers, discussed further below. However, in the absence of such a classifier, hard filter thresholds are a convenient, albeit subjective, trade-off between precision (generally improved by conservative thresholds) and recall (generally decreased by conservative thresholds). If requiring that any of the above thresholds were met in a given VCF, noting that not all VCFs report the relevant information, then 63 % of the total set of false-positive SNPs could be detected. Strikingly, 15 % of the total FPs could only be detected on the basis of proximity to the nearest SNP (Table 1). By contrast, <1 % of the total FPs could only be detected on the basis of the number of reads supporting the variant. Also of note is that all false positives with a read depth ≤5 could also be detected using one or more of the other criteria (Table 1). This serves to re-iterate the above point that these positional characteristics intersect in a complex manner, with some filters overlapping – independently detecting the same FP – to a greater extent than others.

While combining multiple filters using OR logic greatly increases the number of false positives detected, it inevitably discards true-positive calls. In this case, 28 % of the total set of true-positive calls were also discarded when applying one or more of the above filters (Table 1). Consequently, we can consider a ‘value-added’ ratio for each filter: the ratio of the percentage of FP calls removed to the percentage of TP calls also removed, counting only those calls for which this filter can be applied. Higher values indicate that a filter removes proportionately more FPs for each TP (inadvertently) removed, and so is ‘safer’ to use: precision will be increased with less of an effect on recall. On this basis, the ‘safest’ individual filters are those with the highest ratios: up-/downstream mapping bias (ratio 22.4) and the distance to the nearest indel (ratio 18.8) (Table 1). The latter is especially notable: 7.9 % of the total FPs can be discarded using this filter (including 2.7 % that can only be discarded using it), but it only discards 0.4 % of the total TPs (Table 1). This likely reflects the technical difficulty of SNP calling in the vicinity of indels, which requires complex alignment modelling. By contrast, the ‘riskiest’ individual filters to apply are the average quality of the variant-containing reads (ratio 1.9) and variant-call quality (ratio 2.2) (it can be seen in the Supplementary Archive that for many pipelines, the TP and FP distributions show significant overlap for these characteristics). This highlights the fact that false calls can often be made with confidence, ostensibly due to misalignments within the region of the variant (which may be lower-complexity or poorly represented in the reference genome), and that an efficacious means of detecting them is not to look at a single locus but to consider their neighbouring bases. For this reason, proximity to other variant calls is a particularly informative filter.

Aside from the subjective choice of thresholds, other problems with characterizing FPs (and thus, comparing VCF filtering criteria) are that different variant callers populate their VCF with different positional tags, so not all are available to be used as a filter, and that the same tag could be used by different callers without a common nomenclature. For example, depending on caller, ‘read depth’ can refer to an average or absolute count of reads mapped to a given locus, and either include or exclude internally filtered low-quality reads. A description of the positional tags available in each VCF, and used to derive Fig. 2, is given in Table S4. This table also highlights those variant callers that report more data per call, which in principle maximizes the opportunities to detect FPs. For example, 6 % of the total FPs could only be detected on the basis of a disproportionate number of reads mapping up- or downstream of the variant (Table 1), information that is only reported by Freebayes (and pipelines which use it). Although this filter has the highest ‘value-added’ ratio (Table 1), it is limited to a few of the pipelines used in this study. Similarly, a strand bias filter, which requires a minimum proportion of reads mapping to both the forward and reverse strand, has a value-added ratio of 16.1, although this information is not reported by many of the higher-performing pipelines, including Snippy and those employing DeepVariant and GATK.

On the basis of our previous evaluation [5], we anticipated that certain variant callers would be particularly high-performing in these simulations and would by definition produce few, if any, false-positive calls. Consequently, we required a large number of VCFs, spanning a broad range of pipelines – that is, that variant caller paired with multiple different aligners. Although this generated a large set of false-positive calls, it is important to re-iterate that the precision of each pipeline (and thus, the set of false-positive positions analysed above) had to be established in the absence of any VCF post-processing. In general, variant callers make one of two design decisions when producing a VCF: to list a maximal number of variants with the expectation the user will perform their own filtering (the approach taken by, for example, SolSNP), or to list only those variants that pass internal quality control filters, of varying stringency (either by not reporting those that do not pass, or by including them in the VCF with the ‘PASS’ flag not set, the two approaches employed, for example, by Snippy and DeepVariant, respectively). As a consequence, the precision values shown in Table S3 reflect negatively on those variant callers that expect the user to filter their output as a matter of course. This was most notable for SolSNP, Pilon and VarScan, all of which reported positions that passed only a comparatively basic set of internal quality filters; pipelines employing these callers generally had lower average precision (Fig. S1). This has two practical consequences for this study: firstly, the above results should not be taken as a comparative performance evaluation as not all pipelines were run under ‘intended use’ conditions (having no VCF post-processing), and secondly, the overall set of false-positive calls could disproportionately contain those from poorer-performing pipelines (although these may only be considered poorer-performing because their output required post-processing).

As such, we tested whether the distributions shown in Fig. 2 were affected by a disproportionate number of FPs contributed by the poorest-performing pipelines. It is important to note, therefore, that when repeating the analysis after restricting data only to those pipelines with F-score >0.95, quantitatively similar distributions were seen and identical filtering thresholds could be empirically derived (illustrated in Fig. S3 and representing 17 645 245 biallelic true positive and 846 088 biallelic false-positive calls, 75 and 55 % of the original totals, respectively). Furthermore, the set of filters showed similar performance, collectively capturing the same total fraction of FPs (63 %) and with the greatest unique detection rate (19 %) based on proximity to the nearest SNP (although filtering on this basis would also remove the greatest number of TPs; Table S5). With data from the lower-performing pipelines removed from consideration, the ‘value-added’ ratio of several filters greatly increases (Table S5). This was particularly apparent for read depth, the number of reads supporting the variant, and up-/downstream mapping bias, each of which showed a ratio of approx. 60, i.e. they correctly removed 60 times as many FPs for each TP erroneously removed. Strand bias, proximity to the nearest indel, and variant allele frequency also had ‘value-added’ ratios of approx. 20. Explaining these high ratios, we found that with the higher-performing pipelines – which by definition report a greater proportion of true calls with confidence – five of the nine filters removed only a fraction of 1 % of the TPs (the exceptions were variant-call quality, average quality of a variant-containing read, distance to the nearest SNP, and variant allele frequency; Table S5). Taken together, these results suggest that VCF post-processing cannot unilaterally improve the output of all pipelines and that the appropriate course of action is to consider choice of pipeline in conjunction with choice of filter criteria. The data support the use of pipelines which make relatively few false calls to begin with, such as Breseq and Snippy (Fig. 1). Notably, pipelines which make few false calls often apply their own internal criteria, sometimes under user control. For example, the default parameters of Snippy are to require a minimum read depth of 10 and a variant-call quality of 100, and for this reason the aforementioned filters have no added value (see Supplementary Archive).

### Empirical characterisation of true- and false-positive indel calls

Indel calling is technically more challenging than SNP calling, and not attempted by several of the pipelines employed in this study (LoFreq, SNVer, SolSNP and VarScan). To that end, indel-calling F-scores were generally lower than SNP-calling F-scores. Of the set of 990 VCFs, 710 of which contained indels, only 229 (23 %) called indels with an F-score >0.8 (Table S3), as illustrated in Fig. S4 (comparable plots of precision and recall are given in Figs S5 and S6, respectively). It is notable that Snippy, one of the highest-performing SNP-calling pipelines, in terms of F-score, is also the highest-performing indel-calling pipeline (Fig. S4).

**Fig. 3.**
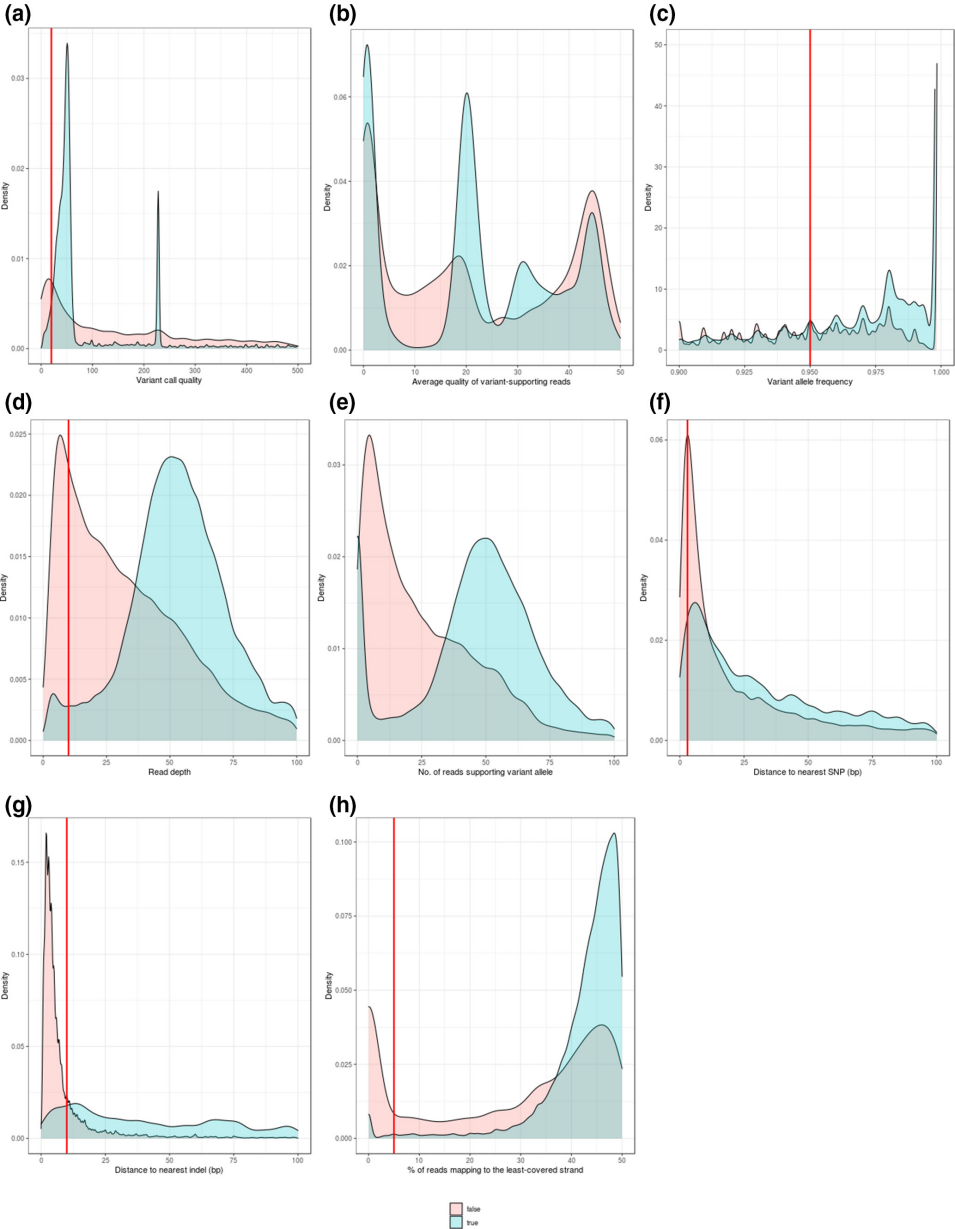
Characteristics of biallelic true and **false-positive** indels. This figure shows data sourced from 990 VCFs, and represents 293 195 biallelic true-positive and 135 295 biallelic false-positive calls. Density plots show the distribution of eight characteristics for the true (blue) and false (red) calls: (a) variant-call quality, (b) average quality per variant-supporting read, (c) variant allele frequency, (d) depth (total number of reads mapped at that locus), (e) number of reads supporting the variant allele, (f) distance to nearest SNP, (g) distance to nearest indel, and (h) percentage of reads mapping to the least-covered strand. Red lines indicate potential hard filter criteria, empirically suggested, and not applicable to plots B and E. These criteria would discard SNPs that have: (a) variant-call quality ≤20, (c) variant allele frequency ≤0.95, (d) read depth ≤10, (f) distance to nearest SNP ≤3 bp, (g) distance to nearest indel≤10 bp, and (h) ≤5 % of reads mapping to the least-covered strand. A summary of the number of false-positive indels detected using these thresholds is given in Table 1. A version of this figure restricted only to pipelines with F-score >0.8 is available as Fig. S7, showing quantitatively similar distributions with identical empirically derived filters. The set of distributions per pipeline are given in the Supplementary Archive.

The full set of 990 VCFs contained a total of 463 386 indel calls, of which 246 852 (53.2 %) were FP (Table S3). For the purpose of characterizing the different attributes of TP and FP calls, we first restricted analysis only to biallelic indel calls. This reduced the dataset to 293 195 biallelic calls, of which 135 295 (46.1 %) were FP. If further restricting the data only to those pipelines with indel-calling F-score >0.8, there were 80 621 biallelic calls, of which 14 303 (17.7 %) were FP. Characteristics of the full set of TP and FP indel calls, and the subset of calls from higher-performing (F-score >0.8) pipelines, are shown in Figs 3 and S7, and summarized in Tables 1 and S5, respectively. As with the analysis of SNP calls, both sets of distributions were quantitatively similar to each other. Furthermore, the distributions were broadly similar to those of the FP SNP calls, although several differences were notable: TP indels have generally lower call quality than TP SNPs (Figs 3a and S7a), both TP and FP indels have overlapping ‘quality by depth’ distributions, so unlike SNP calls no obvious filter exists that may be easily distinguish the two (Figs 3b and S7b), and while FP indels have generally lower read depths than FP SNPs (Figs 3d and S7d), a high proportion of TP indels are supported by few variant-containing reads (Figs 3e and S7e) and for this reason, no filter could be applied on this basis either. It is important to re-iterate that these filters are derived from distributions containing pooled data, and that pipeline-specific distributions may differ; see Supplementary Archive. Most strikingly, and irrespective of whether pooled or pipeline-specific data was considered, FP indels were disproportionately more likely to be close to another indel (Figs 3g, S7g and Supplementary Archive). Unlike SNP calls (Fig. 2i), no data was available for the number of reads mapped up- or downstream of an indel. On the basis of these distributions, different generic filter criteria may be suggested for indels as for SNPs, in particular a higher minimum read depth (10 rather than 5) and a lower minimum call quality (20 rather than 30) (Figs 3 and S7), although as with the SNP distributions, pipeline-specific considerations should apply when choosing filters (characteristics per pipeline are given in the Supplementary Archive). The most informative filter criteria, in terms of the percentage of FP calls uniquely detected, also differed between SNPs and indels. In the case of indels, these were proximity to the nearest indel and variant allele frequency, which by themselves could detect – using the full dataset – 64 and 38 % of the total FP indels to which these filters could be applied, respectively (Table 1). These values were reduced to 44 and 30 %, respectively, when restricting the data only to higher-performing (F-score >0.8) pipelines, although these filters nevertheless remained the most informative (Table S5). It is worth highlighting that both the SNP and indel distributions suggest that a particularly effective means of identifying a false call is by proximity to another SNP or indel, respectively.

### Recommendations to reduce false-positive variant calls

The results of this study complement the best practices literature on variant calling in microbial genomes [59]. We have previously shown that dissimilarity between the reads and the reference genome has a significant impact on the number of false-positive calls, irrespective of variant-calling pipeline [5], and so the simplest means of reducing false-positive calls is to use one of the consistently higher-performing pipelines in conjunction with a reference minimally divergent from the source of the sequenced reads (to that end, some pipelines, notably Breseq [41], estimate sequence divergence and warn the user if it is relatively high). However, should an appropriate reference genome be unavailable, or the sequenced reads be of lower-depth or poorer quality – circumstances that are neither uncommon nor wholly avoidable – it is reasonable to believe that even the highest-performing pipeline would generate additional errors. As such, routinely filtering VCFs to discard potential false-positive calls is prudent. Nevertheless, while hard filters are effective instruments, they are crude ones. Overly stringent VCF filter criteria would generate a disproportionate number of false-negative calls from true positives, given the overlap between the FP and TP distributions (Fig. 2). An optimal set of VCF filters must therefore balance the number of false positives detected against the number of false negatives introduced. We do not attempt to solve this problem here, noting instead that the division of a set of variants into TPs and FPs is a binary classification task, amenable to solution by, for instance, a support vector machine (SVM). While in eukaryotic systems there has been movement away from the routine use of hard filters in favour of machine-learning approaches to VCF filtering (for instance, SNPSVM [58] and GATK VQSR [30, 31]), these are not yet readily applicable to bacteria, given their smaller number of variants, considerable genomic diversity, and the lack of curated variant truth sets. It is in principle possible that for a given genome and pipeline, a SVM can be trained, stored and re-used in order to classify variants in subsequent VCFs. For this purpose, variants which fail multiple hard filters may be used as negative examples for training, as in [60].

In the absence of a machine-learning solution, we have taken an empirical approach to the problem of filter selection, characterizing the distributions of TP and FP SNP (Figs 2 and S3) and indel (Figs 3 and S7) calls across a broad range of pipelines (see also Supplementary Archive). We re-iterate that the purpose of doing so was not to optimize thresholds but to call attention to those filters of greatest apparent efficacy in bacteria and the pipelines to which they may most usefully be applied. Hard filter criteria can be applied using, for instance, the ‘filter’ module of BCFtools [33], with parameters detailed in Table S5. To apply the ‘proximity’ filters, the BCFtools ‘filter’ parameters -g (--SnpGap) and -G (--IndelGap) remove SNPs and indels, respectively, within a certain number of bases of an indel. A similar function is performed by the VCFtools [61] parameter --thin, which removes all sites from a VCF within a given distance of any other. However, our results suggest different thresholds are generally appropriate for removing calls within a certain distance of a SNP or an indel (Figs 2 and 3 and Supplementary Archive). As such, to remove SNPs within, for example, 3 bp of another SNP and 10 bp of another indel, we could first apply VCFtools --thin 3, and then apply BCFtools ‘filter’ --G 10. Although proximity to another variant is one of the most prominent characteristics of a FP, it would be prudent to apply ‘thinning’ operations after all other filters have been used. This would minimize the number of TPs discarded on the basis of a neighbouring FP, provided that FP would also be discarded on the basis of another characteristic (which, as Table 1 indicates, is likely).

It is important to note, however, that many variant callers do not allow certain filters to be applied, as the requisite information is not included in the VCF. The ‘both strands’ and ‘either side’ filters, for example, require variant-containing reads to map on both strands, or to map both up- and downstream of the variant, respectively, but of the 17 distinct VCFs produced in this study, only two contain it – those produced by Freebayes and a pipeline which employs it, SpeedSeq. As such, ones choice of filter criteria cannot be entirely disentangled from the choice of variant caller, and so these recommendations dovetail with those of our previous performance evaluation [5].

We can also conclude from the distributions in Fig. 2 (and the Supplementary Archive) that there are two broad classes of FP SNP. The first, and largest, class comprise data-deficient loci: false calls made on the basis of insufficient data. As shown in Fig. 2, these manifest as peaks in the FP distributions towards the lower end of their range, with FPs easily detected by applying minimum thresholds of depth, quality, proximity to another variant, or the number of reads mapped to both strands or to the left and right of the variant. This class of FPs is the focus of this paper. By contrast to data-deficient loci, however, a smaller class of FPs may be considered ‘data-surfeit’ loci. These positions are supported by a large number of reads, have correspondingly high quality scores, and at face value appear to be confident predictions – the evidential basis for the call seems unambiguous. This is especially apparent in Fig. 2d, where a number of FP calls have depths far exceeding the majority of TPs (this is particularly apparent for the read depth distributions generated by mpileup; see Supplementary Archive). These FPs would not be detected using ‘minimum threshold’ filters, and are perhaps more likely to represent errors in the reference genome. For instance, if the sequenced genome had multiple copies of a region which only appears once in the reference, reads from disparate copies could only be aligned to the single copy in the reference. A mutation in any of these copies would, relative to the deficient reference, be falsely called a SNP. This is a particular consideration for bacteria as they are characteristically diverse, with the alignable fraction of any two genomes from a given species often rather low [62]. We make no attempt to formalize a definition that discriminates between these broadly sketched ‘data-deficient’ and ‘data-surfeit’ classes, noting only that ‘minimum threshold’ filters are not practically applicable to the latter, and that the error is likely not with the reads but the reference. While it would be possible to apply a ‘maximum depth’ threshold – for example, on the basis of coverage being a number of sigma greater than the mean (read depth following a negative binomial distribution [63]) – it may perhaps be more reasonable to consider an alternative reference instead. While the choice of a single (close) reference genome for variant calling is a pragmatic one, alternative methodologies will likely mitigate these reference-associated sources of error. For example, a recent tool, Pandora, takes a pan-genomic approach to variant calling by approximating a reference as a mosaic of known genomes, an approach superior to simply aligning reads to the closest RefSeq genome [62]. Pan-genomic references will also inevitably mitigate the number of FN calls – no single reference genome contains all variants – and although FNs have not been an aspect of this study, it is worth noting that they may have greater impact on data interpretation than FPs. For example, if predicting antibiotic resistance from sequencing data, omitting a single critical SNP may alter the conclusion. In such circumstances, overly stringent VCF filter criteria would be detrimental.

Irrespective of methodology, we are not in a position to suggest optimal filter criteria for all possible pipelines or analytic requirements, and so – given there is no such thing as a ‘universal filter’ (see Supplementary Archive) – limit ourselves only to general recommendations for bacterial short-read variant calling. To that end, on the basis of these results, we would filter SNPs and indels separately, and pay particular attention to a ‘proximity to nearest indel’ filter: irrespective of pipeline, a disproportionate number of FP calls are found in the vicinity of indels (see Supplementary Archive). We also suggest it would be preferable to use a variant caller which is generally higher-performing (for instance, on the basis of this as well as our previous study [5]), calls both SNPs and indels (in the absence of which, proximity filters cannot be satisfactorily applied), and reports only higher-quality positions, thereby not requiring much in the way of hard filtering to begin with. As shown in Table 1, hard filtering cannot discard errors without also making them, so it is arguably better to use a pipeline which reports few errors in the first place than to catch errors already made. It is also clear from the distributions in the Supplementary Archive that filters cannot be universally applied – that is, routinely used irrespective of pipeline – as each pipeline has a distinct error profile, with those making fewer errors not necessarily showing a clear distinction between TP and FP on the basis of a given characteristic (for example, the variant allele frequency of all FPs reported by Breseq, Freebayes and Snippy is 1; see Supplementary Archive). Of the pipelines analysed here, our results suggest that the most straightforward way of minimizing false positives would simply be to use Snippy.

## Supplementary Data

Supplementary material 1

Supplementary material 2
