# Supplementary material for: Generalizable characteristics of false-positive bacterial variant calls
Source: Microb Genom. 2021 Aug 4;7(8):000615. doi: 10.1099/mgen.0.000615 (PMC8549357; doi:10.1099/mgen.0.000615)
Supplement: Supplementary material 1 [file mgen-7-0615-s001.pdf]

Supplementary Figures

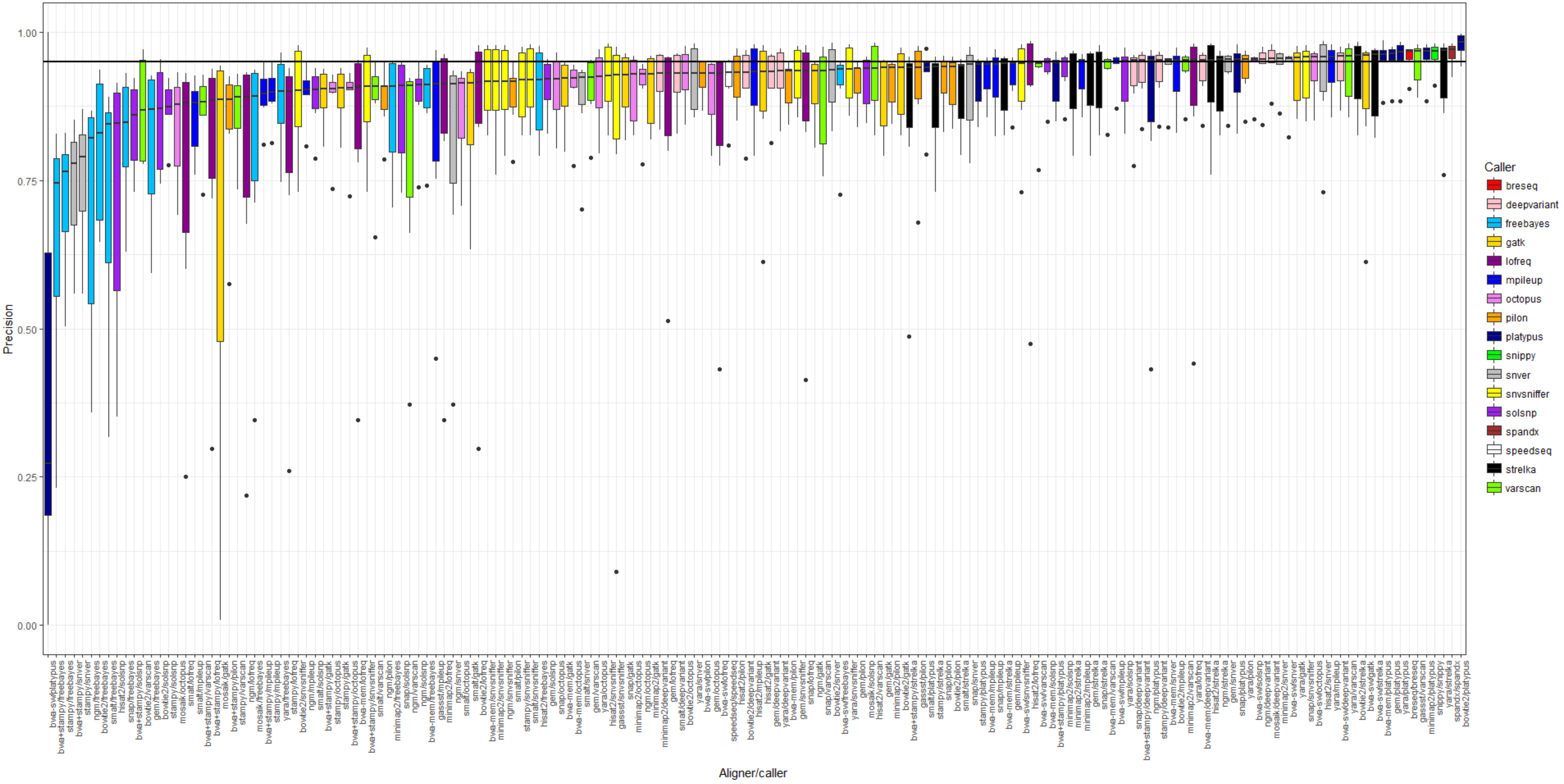

Supplementary Figure 1. Median precision for 166 SNP calling pipelines.

Boxes represent the interquartile range of precision, with midlines representing the median. Upper and lower whiskers extend, respectively, to the largest and smallest values no further than 1.5x the interquartile range. Data beyond the ends of each whisker are outliers and plotted individually. Pipelines are ordered

according to median precision and coloured according to the variant caller employed. The performance metrics for each pipeline, from which this figure was generated, are shown in **Supplementary Table 3**. The line y = 0.95, denoting particularly high precision, is marked.

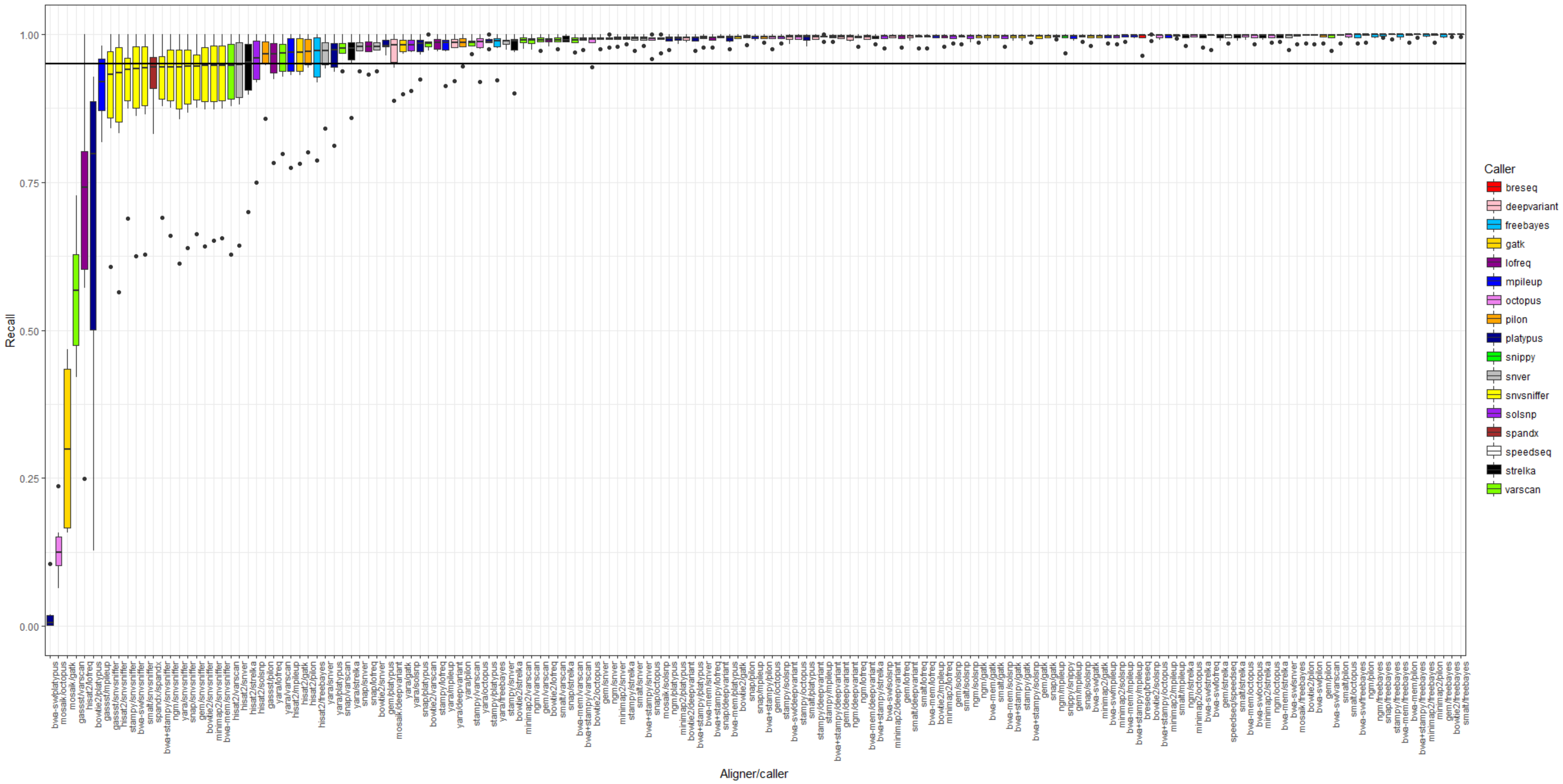

Supplementary Figure 2. Median recall for 166 SNP calling pipelines.

Boxes represent the interquartile range of recall, with midlines representing the median. Upper and lower whiskers extend, respectively, to the largest and smallest values no further than 1.5x the interquartile range. Data beyond the ends of each whisker are outliers and plotted individually. Pipelines are ordered according to

median recall and coloured according to the variant caller employed. The performance metrics for each pipeline, from which this figure was generated, are shown in **Supplementary Table 3**. The line y = 0.95, denoting particularly high recall, is marked.

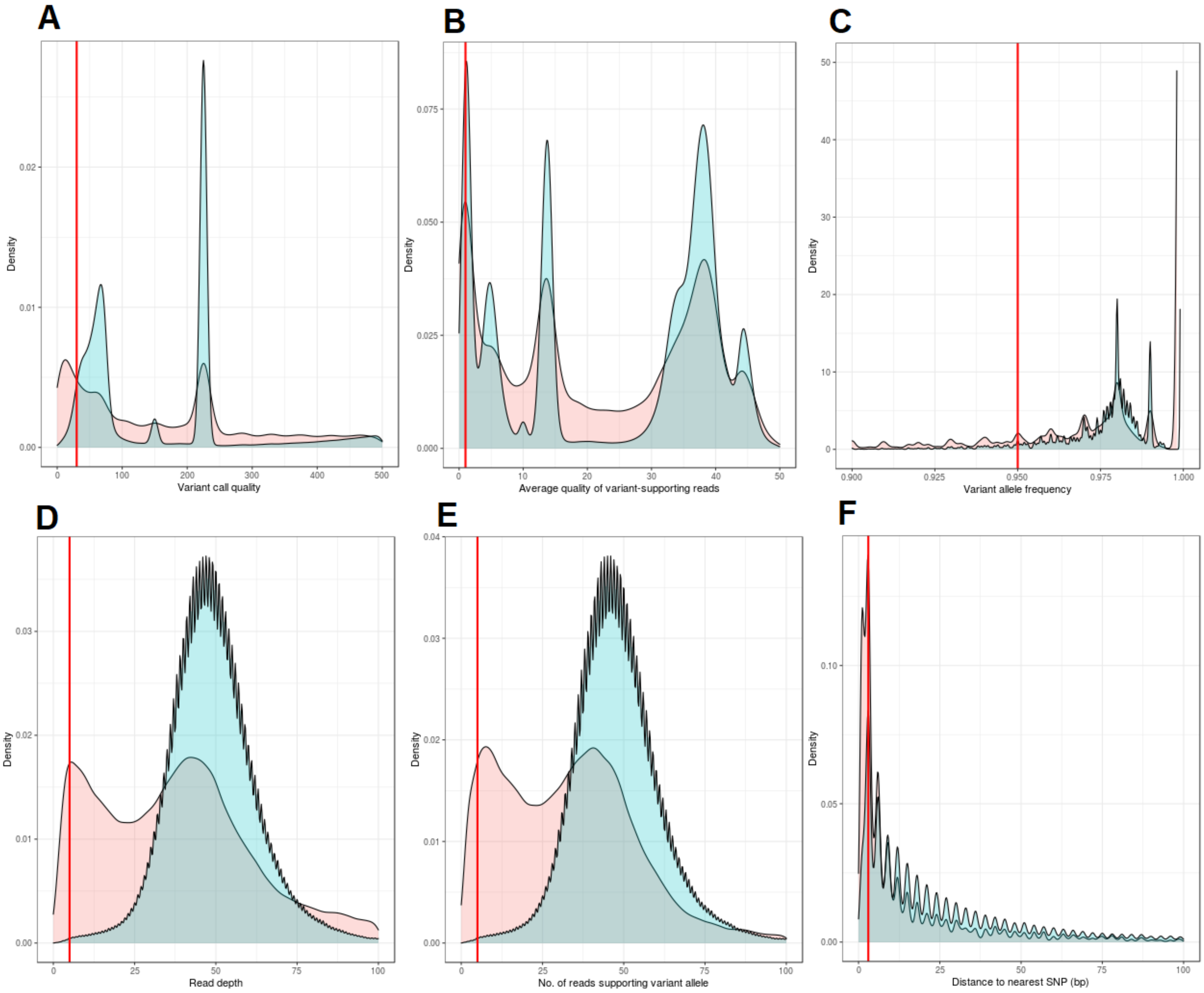

A
Density
Variant call quality
B
Density
Average quality of variant-supporting reads
C
Density
Variant allele frequency
D
Density
Read depth
E
Density
No. of reads supporting variant allele
F
Density
Distance to nearest SNP (bp)

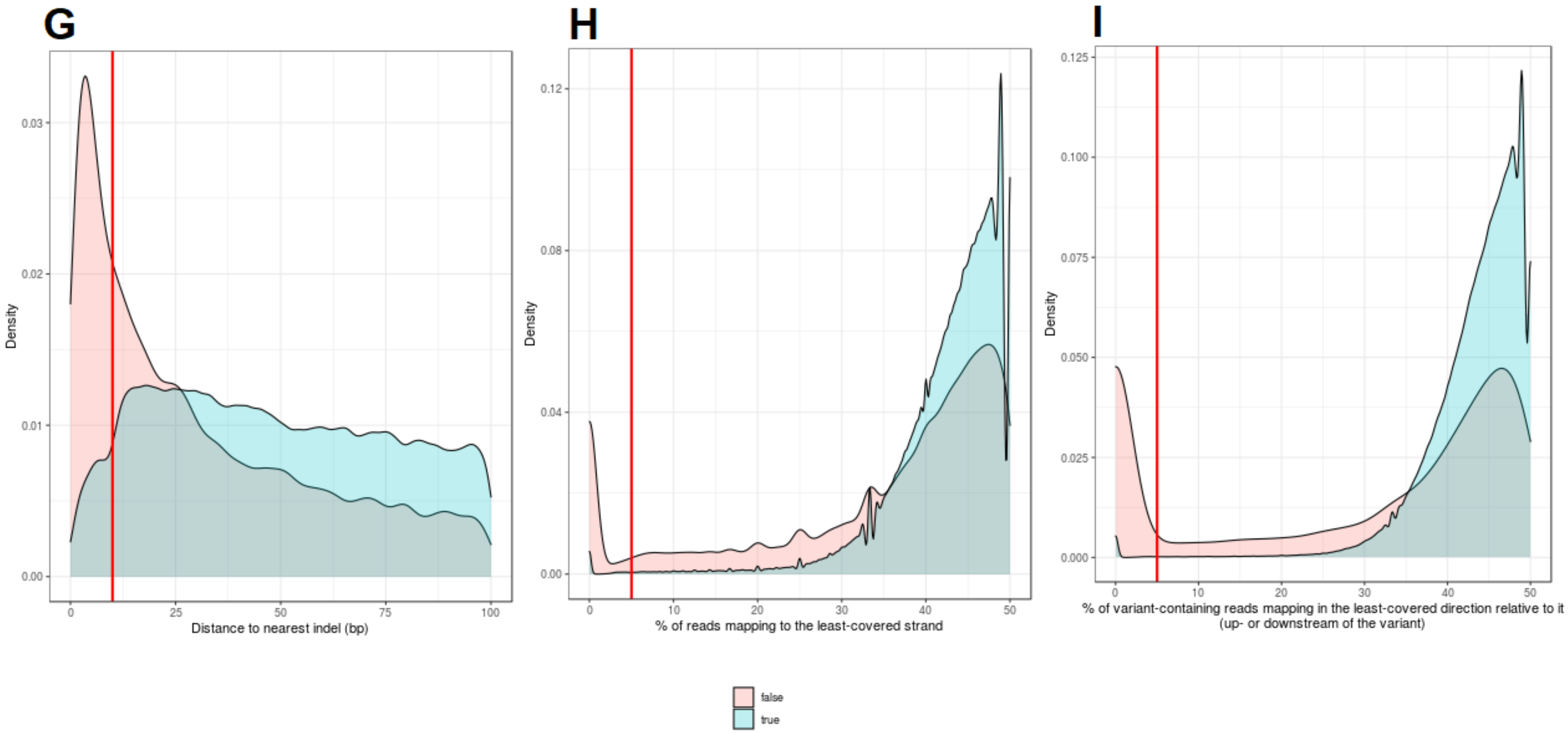

Supplementary Figure 3. Characteristics of biallelic true and false positive SNPs, restricted only to pipelines with SNP calling F-score > 0.95.

This figure shows data sourced from 479 VCFs (those which call SNPs with F-score > 0.95), and represents 17,645,245 biallelic true positive and 846,088 biallelic false positive calls. Density plots show the distribution of nine characteristics for the true (blue) and false (red) calls: (a) variant call quality, (b) average quality per variant-supporting read, (c) variant allele frequency, (d) depth (total number of reads mapped at that locus), (e) number of reads supporting the variant allele, (f) distance to nearest SNP, (g) distance to nearest indel, (h) percentage of reads mapping to the least-covered strand, and (i) percentage of SNP-containing reads mapping in the least-covered direction away from it (in the latter two cases, 50% indicates the variant is equally supported by reads on both the forward and reverse strand, and by reads mapping both up- and downstream of the variant, respectively).

Red lines indicate potential hard filter criteria, empirically suggested. These criteria would discard SNPs that have: (a) variant call quality ≤ 30, (b) average quality per variant-supporting read ≤ 1, (c) variant allele frequency ≤ 0.95, (d) read depth ≤ 5, (e) number of reads supporting the variant allele ≤ 5, (f) distance to nearest

SNP ≤ 3 bp, (g) distance to nearest indel ≤ 10 bp, or ≤ 5% of reads mapping to (h) the least-covered strand, or (i) in the least-covered direction (i.e. the variant is not proportionately supported by reads on both the forward and reverse strand, or both up- and downstream). A summary of the number of false positive SNPs detected using these thresholds is given in **Table 1**. Note that data from SolSNP is not shown in plot A. This is because SolSNP does not follow the VCF specification for defining QUAL continuously, instead capping QUAL at 30. Note also that the 'read depth' distributions in plots D and E are not smooth because different variant callers calculate read depth differently, with some reporting absolute values and others an average (detailed in **Supplementary Table 4**). A version of this figure showing data from all pipelines, irrespective of F-score, is available as **Figure 2**, showing quantitatively similar distributions with identical empirically-derived filters. The set of distributions per pipeline are given in the **Supplementary Archive**.

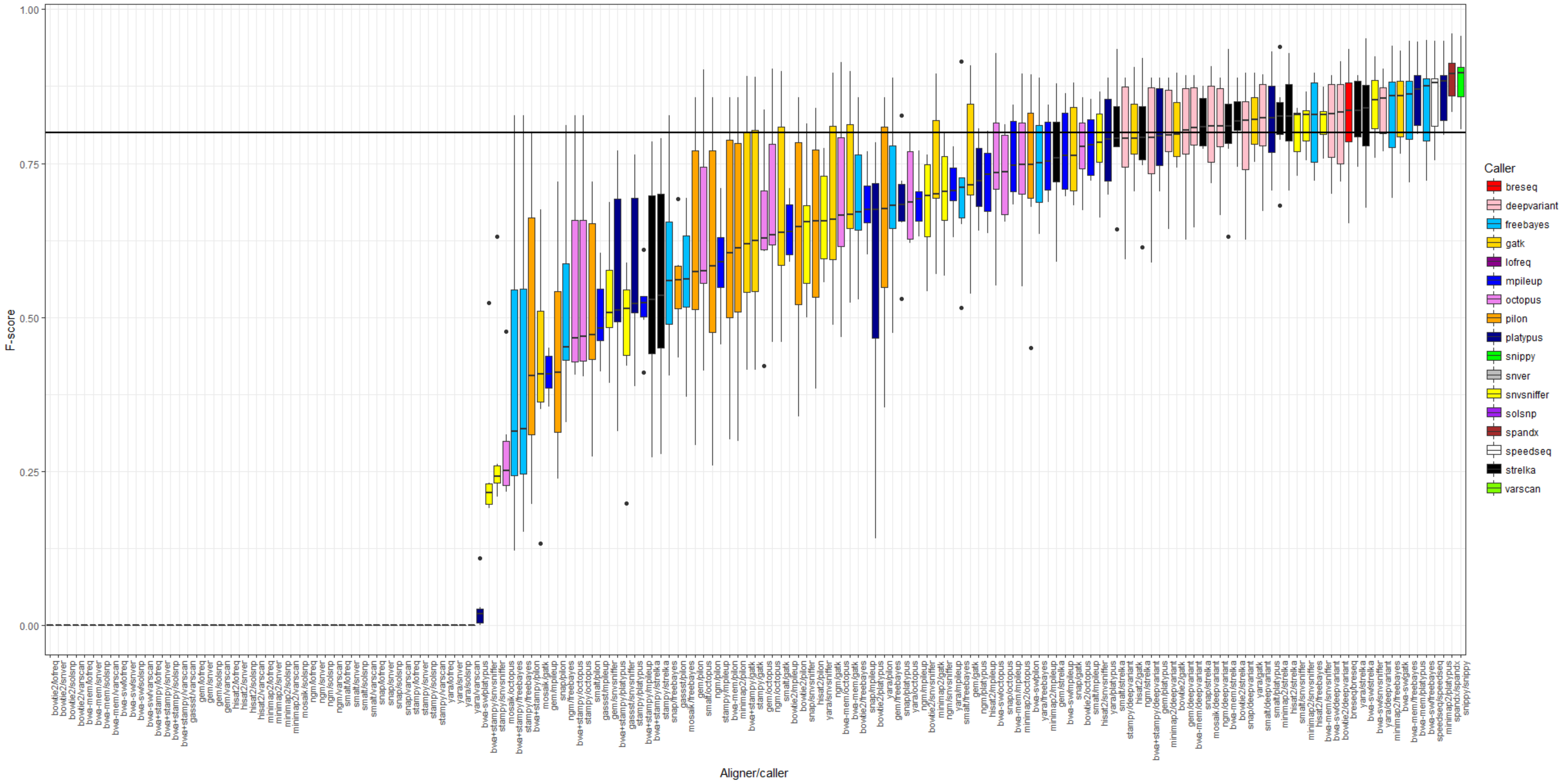

Supplementary Figure 4. Median F-score for 166 indel calling pipelines.

Boxes represent the interquartile range of F-score, with midlines representing the median. Upper and lower whiskers extend, respectively, to the largest and smallest values no further than 1.5x the interquartile range. Data beyond the ends of each whisker are outliers and plotted individually. Pipelines are ordered

according to median F-score and coloured according to the variant caller employed. The performance metrics for each pipeline, from which this figure was generated, are shown in **Supplementary Table 3**. The line y = 0.8, denoting particularly high F-score, is marked.

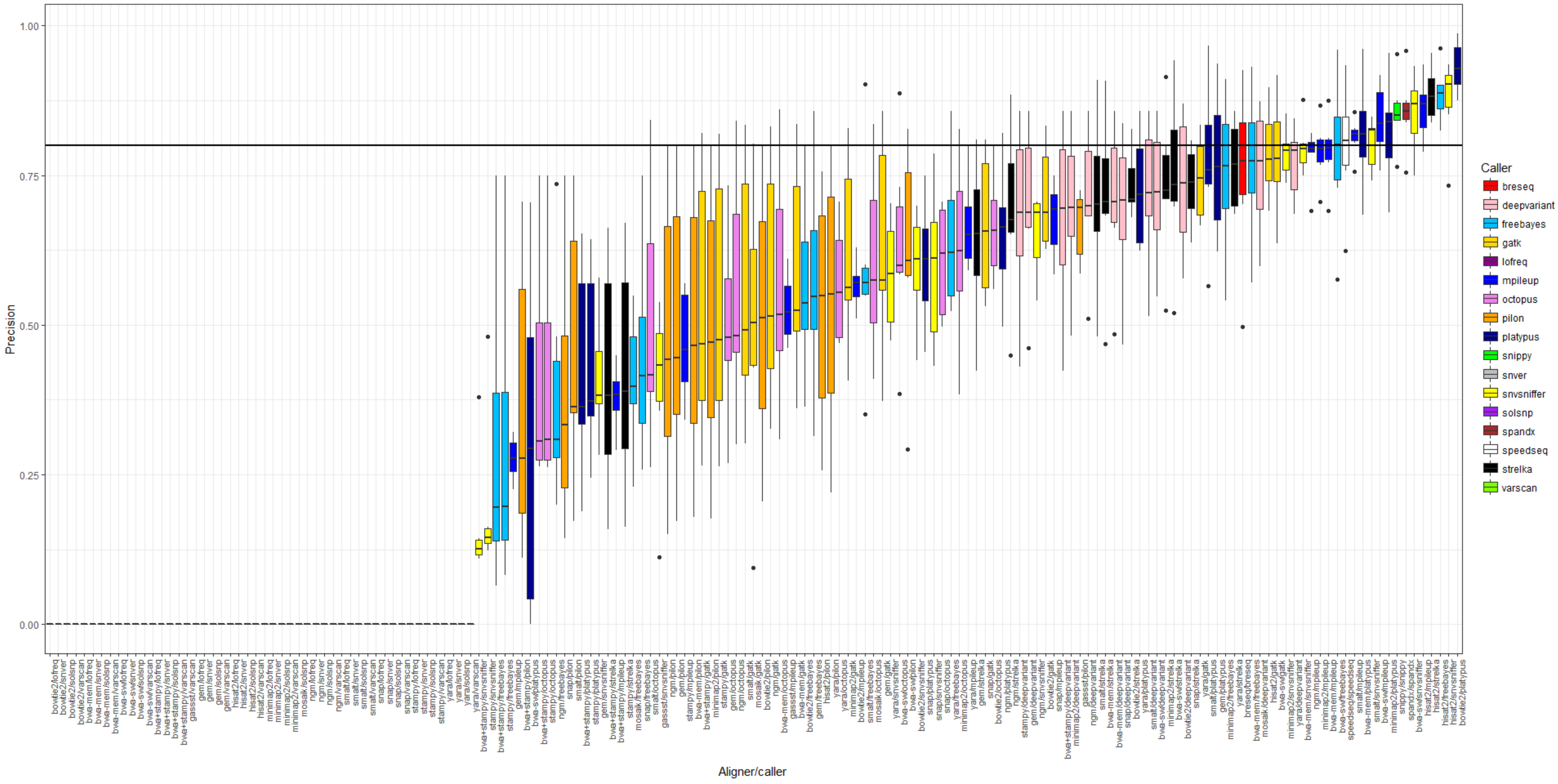

Supplementary Figure 5. Median precision for 166 indel calling pipelines.

Boxes represent the interquartile range of precision, with midlines representing the median. Upper and lower whiskers extend, respectively, to the largest and smallest values no further than 1.5x the interquartile range. Data beyond the ends of each whisker are outliers and plotted individually. Pipelines are ordered

according to median precision and coloured according to the variant caller employed. The performance metrics for each pipeline, from which this figure was generated, are shown in **Supplementary Table 3**. The line y = 0.8, denoting particularly high precision, is marked.

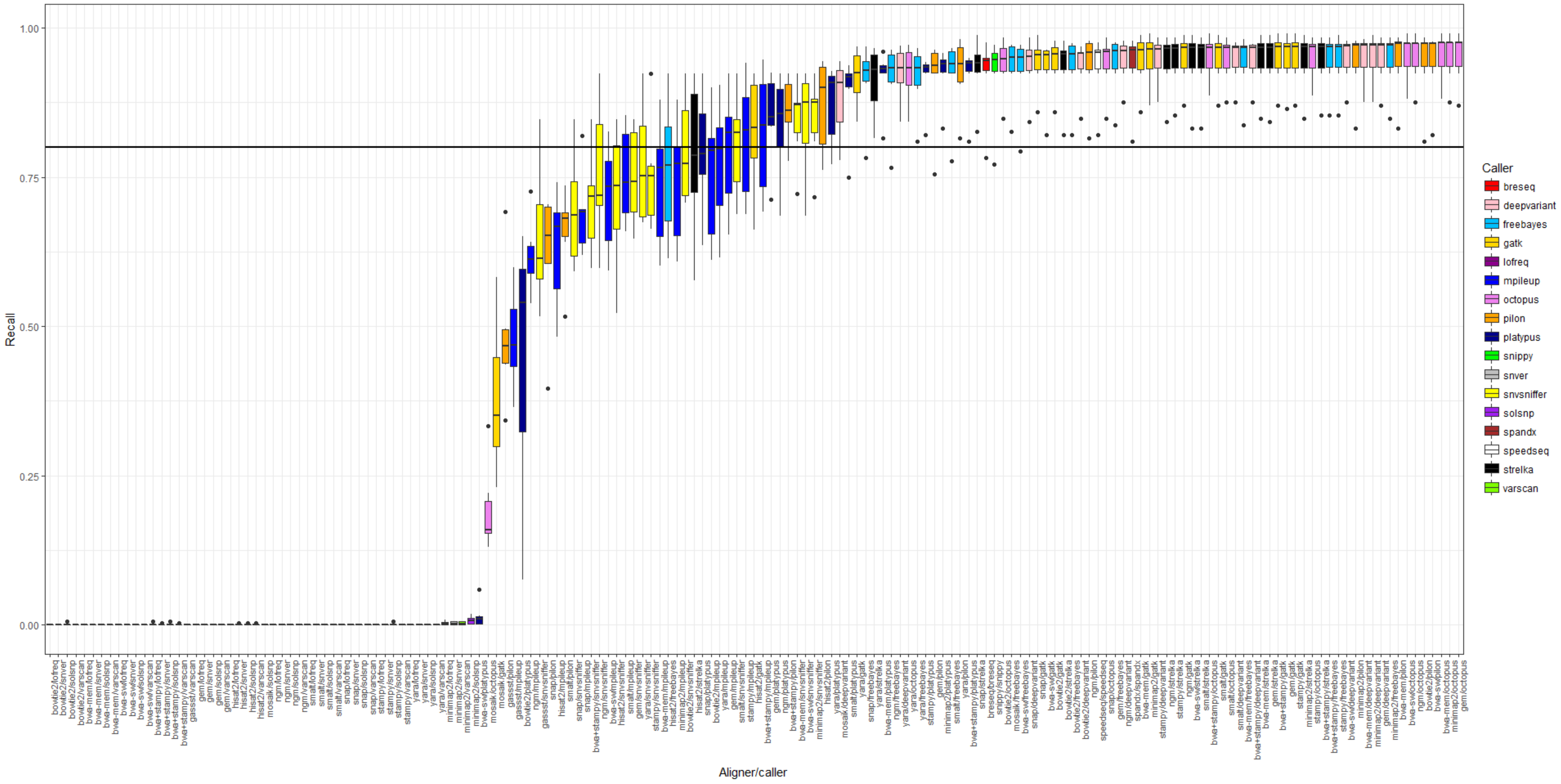

Supplementary Figure 6. Median recall for 166 indel calling pipelines.

Boxes represent the interquartile range of recall, with midlines representing the median. Upper and lower whiskers extend, respectively, to the largest and smallest values no further than 1.5x the interquartile range. Data beyond the ends of each whisker are outliers and plotted individually. Pipelines are ordered according to

median recall and coloured according to the variant caller employed. The performance metrics for each pipeline, from which this figure was generated, are shown in **Supplementary Table 3**. The line y = 0.8, denoting particularly high recall, is marked.

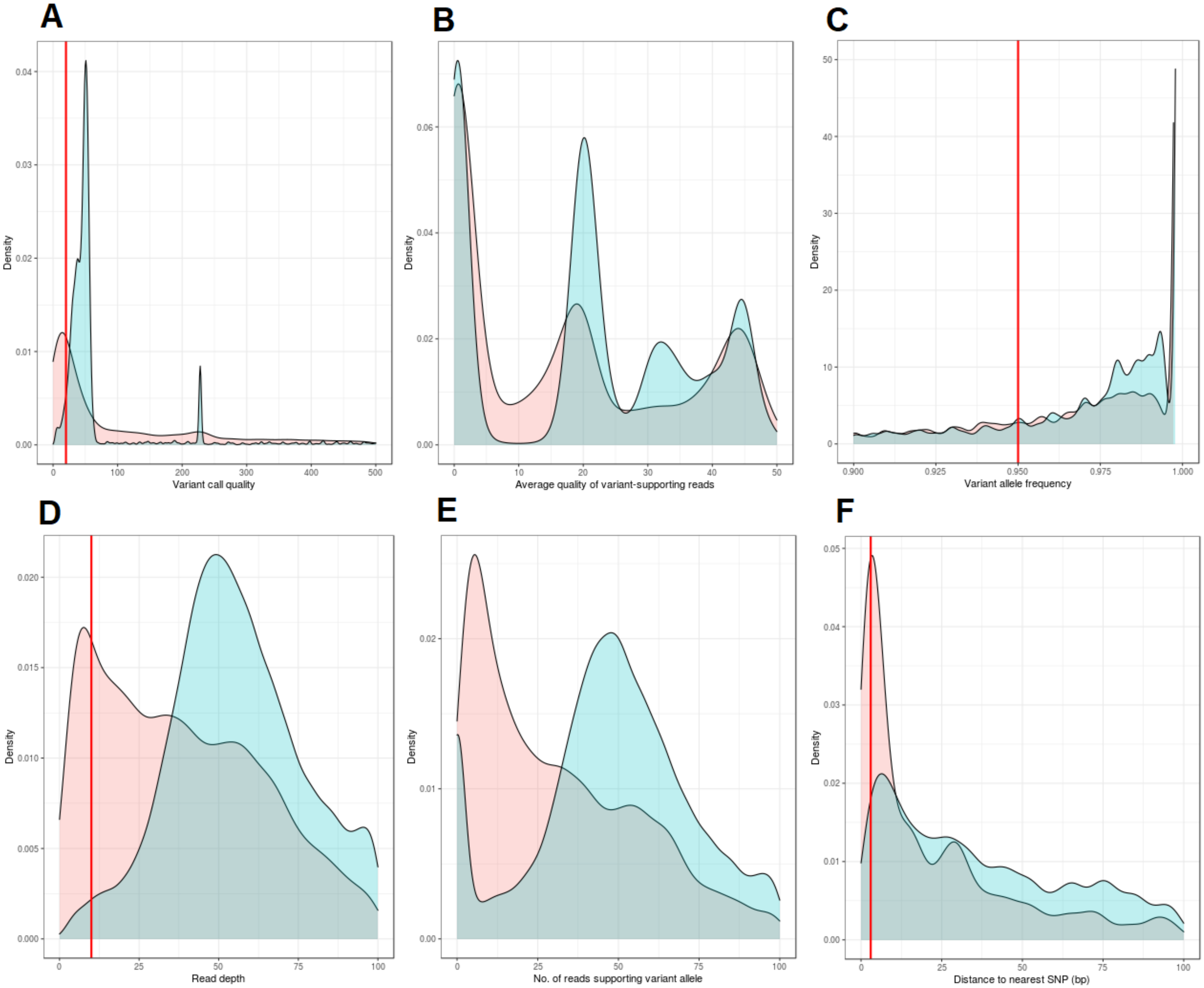
A
B
C
D
E
F
Density
Variant call quality
Average quality of variant-supporting reads
Variant allele frequency
Read depth
No. of reads supporting variant allele
Distance to nearest SNP (bp)

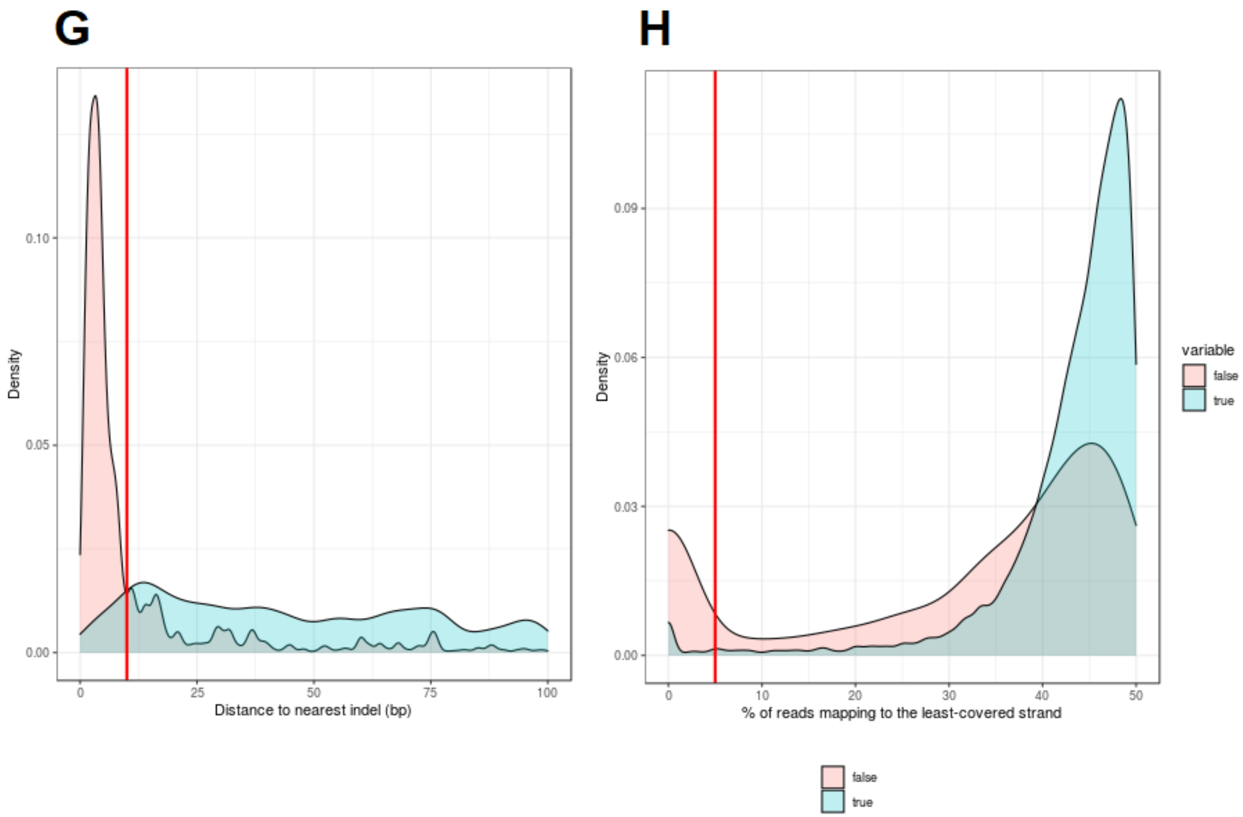

Supplementary Figure 7. Characteristics of biallelic true and false positive indels, restricted only to pipelines with indel calling F-score > 0.8.

This figure shows data sourced from 990 VCFs, and represents 80,621 biallelic true positive and 14,303 biallelic false positive calls. Density plots show the distribution of eight characteristics for the true (blue) and false (red) calls: (a) variant call quality, (b) average quality per variant-supporting read, (c) variant allele frequency, (d) depth (total number of reads mapped at that locus), (e) number of reads supporting the variant allele, (f) distance to nearest SNP, (g) distance to nearest indel, and (h) percentage of reads mapping to the least-covered strand.

Red lines indicate potential hard filter criteria, empirically suggested, and not applicable to plots B and E. These criteria would discard SNPs that have: (a) variant call quality ≤ 20, (c) variant allele frequency ≤ 0.95, (d) read depth ≤ 10, (f) distance to nearest SNP ≤ 3 bp, (g) distance to nearest indel ≤ 10 bp, and (h) ≤ 5% of reads mapping to the least-covered strand. A summary of the number of false positive indels detected using these thresholds is given in **Supplementary Table 5**.

A version of this figure showing data from all pipelines, irrespective of F-score, is available as **Figure 3**, showing quantitatively similar distributions with identical empirically-derived filters. The set of distributions per pipeline are given in the **Supplementary Archive**.
